# Prognostic significance and therapeutic potentials of immune checkpoints in osteosarcoma

**DOI:** 10.17179/excli2021-4094

**Published:** 2022-01-12

**Authors:** Vafa Meftahpour, Ali Aghebati-Maleki, Ali Fotouhi, Elham Safarzadeh, Leili Aghebati-Maleki

**Affiliations:** 1 Immunology Research Center, Tabriz University of Medical Sciences, Tabriz, Iran; 2 Student Research Committee, Tabriz University of Medical Sciences, Tabriz, Iran; 3 Stem Cell Research Center, Tabriz University of Medical Sciences, Tabriz, Iran; 4 Department of Orthopedic Surgery, Faculty of Medicine, Tabriz University of Medical Sciences, Tabriz, Iran; 5 Department of Microbiology, Parasitology, and Immunology, Ardabil University of Medical Sciences, Ardabil, Iran; 6 Department of Immunology, School of Medicine, Tabriz University of Medical Sciences, Tabriz, Iran

**Keywords:** immune checkpoints, osteosarcoma, therapeutic potentials

## Abstract

Although there exist manifold strategies for cancer treatment, researchers are obliged to develop novel treatments based on the challenges that arise. One of these recent treatment approaches is cancer immunotherapy, which enjoys various types of strategies itself. However, one of the most significant methods, in this regard, is employing immune checkpoint proteins (ICPs). Bone sarcomas have several subtypes, with the most common ones being chordoma, chondrosarcoma, Ewing sarcoma, and osteosarcoma. Although many aggressive treatment approaches, including radiotherapy, chemotherapy, and surgical resection, have been employed over the last decades, significantly improved outcomes have not been observed for Ewing sarcoma or osteosarcoma patients. Additionally, chordoma and chdrosarcoma resist against both radiation and chemotherapy. Accordingly, elucidating how recent therapies could affect bone sarcomas is necessary. Checkpoint inhibitors have attracted great attention for the treatment of several cancer types, including bone sarcoma. Herein, the recent advances of current immune checkpoint targets, such as anti-PD-1/PD-L1 and anti-CTLA-4 blockade, for the treatment of bone sarcoma have been reviewed.

## Introduction

As the most frequently occurring malignancy of bone tumor, osteosarcoma (OS) predominantly takes place in young adults and adolescents, with an annual incidence rate of 8.7 per million children for <20 year old individuals, and a 6 % rate among all childhood cancers (Hameed and Dorfman 2011[17]; Izadpanah et al., 2020[23]). The long-term survival rate for individuals with localized OS is 60-80 %, though those with metastases suffer a poorer prognosis immunotherapy in sarcoma (Izadpanah et al., 2019[22]; Mirabello et al., 2009[40]). Unfortunately, it is not exactly clear how the mechanism of OS pathogenesis works. However, it is suggested that several factors, including environmental factors and genetic mutations, are capable of affecting the emergence of OS carcinogenesis (He et al., 2014[19]). 

Since metastatic OS patients suffer from low survival rates, it is essential to develop new therapies, such as immunotherapies that are on the basis of up-regulating the immune responses. One of the main significances of the immune system is the control of tumor (Pourakbari et al., 2020[48]). Thus, appropriately commanding the immune system could offer an efficient therapeutic approach for treating OS. Many studies have emphasized the potential applications of immunotherapy, for instance vaccine therapy and immunomodulation, to eradicate tumors by up-regulating the immune response (Wilky and Goldberg, 2014[69]). Through applying oncolytic virotherapy and adoptive T‑cell therapy, objective responses were obtained in the treatment of OS (Pourakbari et al., 2020[49]). Other potential therapeutic approaches include targeted therapy and immunologic checkpoint blockade. Considerable promises have been suggested through the application of immunotherapy for the improvement of OS outcomes (Liu et al., 2016[35]; Shabani et al., 2019[52]). 

Herein, it has been attempted to review ICPs applicable for OS patients. It is noteworthy to mention that compared to lung cancer, melanoma, and renal cell carcinoma, few research has been conducted on OS patients. Consequently, to generally view such treatment strategy, we additionally include studies performed on other diseases in this review. 

## Biology of the Immune Checkpoint

T lymphocytes possess significant parts in the organization of the immune system. Importantly, such abilities of T lymphocytes for combating malignant cells/invading microorganisms should be regulated through checkpoints capable of preventing the targeting of normal self-tissues (Abdel-Rahman, 2016[1]). It has been known that particular immune-checkpoint pathways are co-opted by tumors as significant mechanisms of immune resistance, especially with regards to tumor-specific T cells. As ligand-receptor interactions initiate several immune checkpoints, they could be regulated via recombinant forms of receptors or ligands or be blocked through antibodies (Pardoll, 2012[46]). 

Therefore, selectively inhibiting such regular inhibitory checkpoints/mechanisms could result in T lymphocyte activation, and subsequently lead to the promotion of more efficient anti-tumor responses (Topalian et al., 2012[61]). Several therapies based on immune checkpoint blockade have been approved by the FDA to treat many tumors (Table 1(Tab. 1)). In fact, realizing long-term responses in patients corresponds to a transformative event. Following the approval of ipilimumab (anti-CTLA-4) by FDA to treat metastatic melanoma in 2011, 5 more checkpoint blockade therapies have emerged, all of which target the PD-1/PD-L1 axis and are used for treating several tumor types. Moreover, to treat advanced melanoma, combination therapy using ipilimumab and nivolumab (anti-PD-1) has been approved, which is more efficient than either of the monotherapies (Wei et al., 2018[68]). 

Being expressed at the level of immune cells throughout their activities, immune checkpoints (ICPs) are molecules of protein that regulate immune cell activity when bound to their ligand, and prevent them from progression in the immune responses as well as damaging normal self-tissues through the inhibition of release of immune cell-produced toxins (Khosroshahi et al., 2021[26]). It has been reported that the ligands of ICPs are expressed at their level to bond with them, resulting in the evasion of the immune system by the tumor (Khosroshahi et al., 2021[26]). Using blockers for these molecules improves the anti-tumor functions of T cells. However, of significance is setting a codified program for proper application, and determining the optimal medication dose for treating the disease.

### CTLA-4

As the first ever receptor of the immune checkpoint targeted clinically, CTLA-4 enjoys exclusive expression on T cells, where it principally modulates the amplitude of the early stages of the activation of T cells. Mainly, the activity of CD28, as a T cell co-stimulatory receptor, is counteracted by CTLA-4. On the condition that cognate antigens first engage TCR, CD28 affects T cell activation. Sharing an almost 30 % identity in amino acids with CD28, CTLA-4 is a member of CD28-B7 immunoglobulin superfamily. TCR signaling is sharply amplified by CD28 signaling upon antigen recognition, which together activates T cells. Identical ligands are shared by CTLA-4 and CD28; CD86 (also known as B7.2) and CD80 (also known as B7.1). Even though how CTLA-4 functions is not exactly understood, and since CTLA-4 enjoys a greater affinity for both ligands, it is suggested that when it is expressed on T cell surfaces, T cell activation is reduced through outcompeting CD28 for binding to CD86 and CD80, along with the active delivery of inhibitory signals to the T cell. 

In fact, through which signaling pathways the activation of T cells is blocked by CTLA-4 is still under debate. However, several researchers have proposed that protein phosphatase activation, for instance PP2A and SHP2 (also known as PTPN11), could be significant in counteracting kinase signals induced by CD28 and TCR. On the other hand, signaling-independent inhibition of T cells is conferred by CTLA-4, by sequestering CD86 and CD80 from the engagement of CD28, as well as actively removing CD86 and CD80 from the surface of the antigen-presenting cells (APCs).

While the expression of CTLA-4 takes place through activated CD8+ effector T cells, its foremost physiological role is supposed to be within affecting two major CD4+ T cell subsets: down-modulating the activity of helper T cells and enhancing the immunosuppressive activity of regulatory T (Treg) cells. The blockade of CTLA-4 leads to an extensive augmentation of helper T cell-dependent immune responses. On the other hand, when engaged on Treg cells, CTLA-4 improves their suppressive functions. Importantly, the forkhead transcription factor (FOXP3) targets the CTLA-4 gene. It is not known through which mechanisms the immunosuppressive function of Treg cells is enhanced by CTLA-4, though Treg cell-specific blockade or knockout of CTLA-4 considerably impedes their ability to control both antitumor immunity and autoimmunity. 

The conventional wisdom that underlies CTLA-4 inhibition is releasing the feedback inhibition of T cells that have encountered antigens. The first efforts in this regard were made in 1996 by James Allison and colleagues. It was revealed that when CTLA-4 antibodies block the activity of CTLA-4 in pre-established tumors in several murine models of tumor, the tumor is rejected. Additionally, immunity against second exposure to tumor cells was provided to animals by the antibodies (Aghebati-Maleki et al., 2019[2]; Liu et al., 2013[33], 2014[34]; Wang et al., 2011[66]). 

Such discovery elevated the interest in this respect, resulting in several clinical trials and the development of Ipilimumab as an entirely human IgG1 anti-CTLA-4 antibody applicable in clinical testing. The success of preclinical studies and clinical trials eventually resulted in the FDA approval of Ipilimumab in 2011. Elevated load of mutations, enhanced pretreatment level of tumor infiltrating lymphocytes, and amplified tumor antigen specific T cells were attributed to improved clinical activity. As the first drug successful in phase III clinical trials for the treatment of late stage metastatic melanoma, Ipilimumab is still prominent for improving patient survival. Moreover, tremelimumab or IgG2 isotype of CTLA-4 antibody has been employed for the treatment of metastatic melanoma, beside ipilimumab which is IgG1 type of anti-CTLA-4 antibody. However, tremelimumab failed to represent satisfying efficacy in phase III clinical trials (Shang et al., 2013[53]). There are still ongoing investigations with regards to tremelimumab, using it as either monotherapy for metastatic mesothelioma or combined with other immunotherapeutics for the treatment of cancers such as liver, gastric, pancreatic, and bladder cancer, as well as squamous cell carcinoma of the head and neck and non-small cell lung cancer. In a recent phase IIb trial, tremelimumab monotherapy did not show survival benefits for metastatic melanomas. So far, there has been no recognized pretreatment biomarker applicable as part of standard-of-care therapeutic decision-making. However, identifying particular post-treatment immune responses, which are seemingly linked to clinical outcomes, has sparked insights. The fact that CTLA-4 is considerably linked to the risk of osteosarcoma and could possess important parts in the carcinogenesis of osteosarcoma has been demonstrated by many meta-analyses (Liu et al., 2013[33], 2014[34]). In comparison to healthy controls (OR 2.27, p=0.010, and OR 1.41, p=0.015), it was shown by Wang et al. that the frequency of the +49A allele and the CTLA-4 +49AA genotype were meaningfully enhanced in osteosarcoma patients. This suggests that the +49G/A polymorphism of CTLA-4 gene is linked to enhanced risk of osteosarcoma (Wang et al., 2011[66]). Additionally, in Chinese Han population, it was shown that the genetic polymorphisms of CTLA-4 could have a potential relation to the risk of OS, and thus could be employed as molecular markers in this regard. Together, these results emphasize the clinical advantages of such therapeutic approach for treating OS. 

It has been demonstrated for OS metastatic patients that the combined anti-CTLA-4 program along with other immune factors with significance in this regard could result in improved anti-tumor activity and prompt recovery from the disease. Nevertheless, additional experiments are required to obtain the best outcomes in this respect. 

### PD-1

PD-1, as another receptor of the immune checkpoints, has emerged as a recent potential target, which emphasizes the variety of immunological manipulations that are molecularly defined and enjoy the ability to induce antitumor immune responses through the immune system of the patient. 

The main role attributed to PD-1, as opposed to CTLA-4, is the limitation of T cell activity in peripheral tissues when there is an inflammatory response against infections, as well as limiting autoimmunity. This is translated into a key mechanism of immune resistance inside the microenvironment of the tumor. When T cells are activated, the expression of PD-1 is induced. Through engagement with a ligand, PD-1 is capable of inhibiting kinases involved in the activation of T cells by the SHP250 phosphatase, even though there could be other induced signaling pathways. Moreover, since the engagement of PD-1 is capable of inhibiting the stop signal of TCR, such pathway could adjust the duration of the contact between T cell-target cell or T cell-APC. The expression of PD-1 takes place highly on Treg cells, which is akin to CTLA-4, enhancing their proliferation when a ligand is present. Blocking PD-1 pathway might further improve antitumor immune responses through reducing the suppressive activity and/or the number of intratumoral Treg cells, since several tumors have a high Treg cell infiltration which presumably suppresses effector immune responses. 

PD-1 ligand 1 or PD-L1 (also recognized as B7-H1 and CD274) and PD-L2 (also recognized as B7-DC and CD273) are the two ligands of PD-1. Sharing a 37 % homology in sequence, these members of the B7 family have arisen by gene duplication, positioning them with a 100 kb distance in the genome. The recent discovery of the molecular interaction between CD80 and PD-L1 led to the understanding that T-cell- or APC- expressed CD80 might be capable of behaving as a receptor instead of a ligand through inhibitory signal delivery when engaged by PD-L1. However, the relevance of such interaction in the immune resistance of the tumor is yet to be defined. Lastly, the genetic evidence of PD-1-deficient T cells puts forward the idea that both PD-L2 and PD-L1 might be capable of binding to a T cell-expressed co-stimulatory receptor. Such complicated binding interactions are similar to CD80 and CD86 ligand pair, which could bind to CD28 as a co-stimulatory receptor expressed on resting T cells as well as CTLA-4 as an inhibitory receptor expressed on activated T cells. Nonetheless, PD-1 mostly controls the activity of effector T cells in tumors and tissues, while CTLA-4 principally controls the activation of T cells. To select recombinant ligands and antibodies for clinical use, it is important to understand the part these various interactions possess in different cancer settings. 

The expression of PD-1 takes place more broadly as compared to CTLA-4, and its induction occurs on other non-T lymphocyte subsets that are activated, such as natural killer (NK) cells and B cells, limiting their lytic activity. Consequently, while blocking PD-1 is often considered to enhance the activity of effector T cells in tissues and the tumor microenvironment, it is possible to assume that it improves the activity of NK cells in tissues and tumors, in addition to enhancing the production of antibodies either by directly affecting PD-1+ B cells or in indirect manners. Since the primary role of PD-1 ligands in cancer is seemingly immune inhibition in the tumor microenvironment, and since PD-1 is capable of inhibiting the function of lymphocytes only when engaged with PD-L1 and PD-L2, it is important to understand the pattern of expression of PD-1 ligands in order to determine whether they would be suitable in therapy if blocked. There exist two known PD-L1 expression types; an adaptive mechanism triggered through stimulating inflammatory cytokines, and an innate mechanism triggered through genetic mutation, with the former being mainly caused by IFNγ (Hameed and Dorfman, 2011[17]). High levels of expression were initially attributed to most samples of lung cancer, ovarian cancer, and melanoma. Later, the upregulated expression of PD-L1 was also reported for several human cancers. For one, 50 % of osteosarcomas show intermediate, and 23.7 % of them show high expression levels in this regard (Shen et al., 2014[55]). While the expression of PD-1 is demonstrated to have a correlation with the progression of the osteosarcomas, higher PD-L1 expression levels in tumor cells are reported to have a positive correlation with TILs in osteosarcoma. Osteosarcoma had 14-75 % higher expression of PD-L1 in tumor tissues, with significant correlation to metastasis and mortality risk, as reported by a recent systematic meta-analysis including 868 total patients from 14 studies (Zhu et al., 2017[74]; Shen et al., 2014[54]). 

As a PD-1 blocking antibody, Nivolumab is considered a targeted therapy. Following nivolumab treatment, partial responses were observed in patients with metastatic sarcoma who had osteosarcoma, epithelioid sarcoma, and dedifferentiated chondrosarcoma (Veenstra et al., 2018[63]). Interestingly, the regulation of the cell growth of osteosarcoma and resistance against paclitaxel and doxorubicin have been attributed to PD-L1 (Liao et al., 2017[31]).

Recently, promising progress has been made through tumor immunotherapy targeting PD-1: PD-L1/PD-L2. However, combining the application of multiple biomarkers is yet to be fully explored. Combining ipilimumab with nivolumab was shown to bring about more survival benefits compared to nivolumab monotherapy. 

It has been shown in preclinical studies on mouse models that the upregulation of immune checkpoints, including TIM-3, takes place as a result of anti-PD-1 antibody treatment, which might lead to resistance against anti-PD-1 antibody therapy. The detectable soluble PD-L1 (sPD-L1) in the blood is a derivative of the alternative variants of the transcripts of PD-L1, which might have a relation to ICBs-mediated anti-tumor response cytokines, including TNF-α, IFN-γ, or IFN-α. As compared to patients with high plasma levels of sPD-L1, patients with low plasma levels of sPD-L1 achieved partial or complete response in a larger proportion. Additionally, the development of the progressive disease in those with low plasma levels of sPD-L1 was observed in a lower proportion. The association of higher levels of sPD-L1 to enhanced tumor grade, larger tumors, and elevated death risk has been reported in clear cell renal cell carcinoma (ccRCC) patients. It is assumed that the immune system of the host is damaged by sPD-L1, thus cancer progression is promoted, reducing the clinical outcomes. Taken together, the plasma level of sPD-L1 might be a valuable biomarker in the prediction of ICBs treatment response. There exist three distinct phase II clinical trials with regards to checkpoint inhibitors on osteosarcoma patients at present; one using anti-PD-1 antibody Pembrolizumab (NCT02301039), and the other two assessing anti-PD-1 antibody Nivolumab without or with anti-CTLA-4 antibody Ipilimumab (NCT02304458 and NCT02500797). Therefore, based on the results of these clinical trials, the efficiency of checkpoint inhibitors could be elucidated in osteosarcoma patients. It has been found that PD-1 percentage is considerably enhanced on both peripheral blood CD8+ and CD4+ T lymphocytes in osteosarcoma patients. Also, the progression of osteosarcoma has been attributed to PD-1 (Zheng et al., 2015[73]). Moreover, high expression level of PD-L1 has been determined by assays based on RNA in tumor samples and human osteosarcoma cell lines. Thus, inhibiting PD-1/PD-L1 could be an interesting approach capable of restoring the function of the immune system against osteosarcoma cells (Koirala et al., 2016[27]; Shen et al., 2014[55]; Wang et al., 2016[67]). It was shown in 2018 that the rate of the response of anti-PD-1was greater compared to that of anti-PD-L1 antibody (Shabani et al., 2019[52]). Since CTLA-4 is expressed by Tregs, obtaining the anti-tumor function of anti-CTLA-4 antibody could be performed through inhibiting CTLA-4 on Tregs in order to reverse the suppression of the activation of T cells (Pardoll, 2012[46]; Topalian et al., 2012[61]). PD-1 expression onTregl surface has been noted by some studies (Liu et al., 2013[33], 2014[34]; Wang et al., 2011[66]; Wei et al., 2018[68]), and the significance of PD-1 on Tregs has been shown (Liu et al., 2014[34]; Shen et al., 2014[55]). Despite the existence of comprehensive studies on the relationship between anti-PD-1 antibody and Treg (Zhu et al., 2017[74]), how anti-PD-1 antibody affects Tregs is yet to be defined. With respect to the use of anti-PD-1 antibody for the treatment of osteosarcoma, there only exist three interim reports on clinical trials (Liao et al., 2017[31]; Shen et al., 2014[54]; Veenstra et al., 2018[63]) and one basic research report (Zheng et al., 2015[73]). Additionally, the mechanism of the expression of PD-L1 is unknown, even though it is reportedly expressed in osteosarcoma (Wang et al., 2016[67]). Its antitumor effects have also been established *in vivo*, through the alterations of the volume of the tumor and overall survival time of following the administration of anti-PD-1 antibody in a subcutaneously implanted mouse model of osteosarcoma. Thus, more studies are required to demonstrate the effect of anti-PD-1 antibodies in osteosarcoma patients.

### TIM-3

As one of the members of the TIM gene family, T-cell immunoglobulin and mucin domain-containing-3 (TIM-3) is found in humans, along with TIM-1 and TIM-4, while TIM-1 through TIM-8 are found in mice (Abdel-Rahman 2016[1]). The TIM family members are located on chromosome 5q33.2 in humans. Among them, the expression of TIM-3 takes place on CD8+ T cells (myeloid lineage cells), Th17, and T helper 1. When engaged with its ligands, TIM-3 is capable of suppressing Th17 and Th1 responses. In tumor tissues, there exists a significant association between the polymorphisms of TIM-3 and PD-1 (programmed cell death 1) and the expression level of TIM-3 and PD-1, such that by administrating PD-1 and TIM-3 synergistic promotion of tumor growth would be observed (Topalian et al., 2012[61]). The inhibitory effects TIM-3 exerts on anti-tumor immunity are highlighted by the above mentioned preclinical studies. 

Four ligands have been attributed to TIM-3, including phosphatidylserine (Epstein and Philip, 1987[13]), high-mobility group protein B1 (HMGB1), carcinoembryonic antigen cell adhesion molecule 1 (CEACAM-1), and galectin-9 (Gal-9), the latter of which was the first to be recognized (Wei et al., 2018[68]). Through negative regulation of T-cell immunity, TIM-3/Gal-9 is capable of inhibiting cancer immunity. The connection between Gal-9 and TIM-3 IgV domain could terminate the immune responses of T helper 1 (Th1). Also, TIM-3 is capable of inhibiting T-cell immune responses, and has been shown to have an association with immune exhaustion, inducing chronic viral infection (Liu et al., 2013[33]). Through the blockade of the TIM-3 pathway, cancer immunity could be enhanced and the production of interferon-gamma (IFN-γ) could be increased in T cells (Wang et al., 2011[66]). The expression of CD8+ TIM-3+ T cells has been shown to have a correlation with PD-1 expression *in vitro *and* in vivo*. Also, compared to TIM-3 negative CD8+ T cells, TIM-3 and PD-1 positive CD8+ T cells generate less IFN-γ 21. Interestingly, the IFN-γ in peripheral NK cells could be increased by using anti-TIM-3 antibodies. Not only could LAG-3, TIM-3, or PD-1 improve the response of T cells to tumor antigens, but they also enjoy a synergistic function (Friedman et al., 2016[15]). The production of cytokines, including IL-2, tumor necrosis factor-alpha (TNF-α), and IFN-γ could be inhibited by TIM-3+ PD-1+ CD8+ TILs. Using the blockade of PD-1 in combination with TIM-3 is more efficacious than either alone (Ishihara et al., 2017[21]). 

Since TIM-3 is expressed on several T cells, it could be a favorable target in cancer (Liu et al., 2014[34]), having significant roles in innate immune cell-mediated antitumor immune responses. PD-1 antibodies have been reported to have a possible role in increasing the expression of TIM-3 in lung cancer in *in vivo* models, demonstrating that TIM-3 could be a marker of PD-1 blocking antibody resistance. However, the role that TIM-3 plays in cancer immunity needs further investigation. In fact, recent treatments targeting TIM-3 might bring about a breakthrough in cancer therapy.

Antitumor immunity could be enhanced by TIM-3 antibodies, since T helper 1 (TH1) cell responses could be inhibited by TIM-3, the ligand of which is galectin 9 (which itself enjoys an upregulation in several cancer types, such as breast cancer). The co-expression of TIM-3 with PD-1 on tumor-specific CD8+ T cells has also been reported, and dually blocking them considerably improves the *in vitro* cytokine production and proliferation of human T cells when stimulated via NY-ESO-1 or the cancer-testes antigen. *In vivo*, coordinately blocking TIM-3 and PD-1 has been reported to be capable of enhancing tumor rejection and antitumor immune responses under the same conditions in which single blocking brought about only modest effects. Increased TIM‐3 on CD8+ T and CD4+ T cells has been reported in the peripheral blood of OS patients, where high levels of TIM-3 had a positive correlation with poor prognosis, pathological tumor fracture, metastasis, and tumor stages (Liu et al., 2016[32]). Notably, the immune suppression is not directly mediated by the expression of TIM-3 in osteosarcoma patients. Instead, the interaction between TIM-3+ T cells and Gal9-expressing CD4+ CD25+ Tregs, naive CD4+ T cells, and monocytes leads to the progressive suppression of the responses of Th1 (Li et al., 2017[30]). In osteosarcoma tissues, the co-expression of TIM-3 with several EMT-related proteins, such as Smad, Snail, Slug, and Vimentin has been documented, which makes a contribution to the enhanced cancer cell invasiveness (Shang et al., 2013[53]). Similarly, the invasion of melanoma cells (B16) is promoted by TIM-3 through the elevation of the activity of NF-κB, which leads to the metastasis of melanoma (Wu et al., 2010[70]). Feng et al. have reported that transfection of MG-63 cells with the siRNA of TIM-3 is capable of inducing reduced expression of vimentin, E-cadherin, and Snail, as well as NF-κB phosphorylation. On this basis, it was assumed that TIM-3 is capable of promoting EMT and inducing the metastasis of osteosarcoma through the activation of the NF-κB/Snail signaling pathway (Feng and Guo, 2016[14]). 

### Lymphocyte-activation gene 3 (LAG-3)

Discovered by Triebel et al almost 30 years ago in a transcript form, the expression of which takes place via a cytokine IL-2-dependent natural killer cell line, LAG-3 encodes a protein similar to co-receptor CD4. In fact, LAG-3 is the third inhibitory receptor pathway targeted clinically. The function of LAG-3 includes the control of excessive activation after persistent exposure to antigens in order to prevent autoimmunity (Koirala et al., 2016[27]; Wang et al., 2016[67]); nonetheless, it is capable of making a contribution to the conditions under which T cells dysfunction in the tumor microenvironment (TME) (Liu et al., 2016[32]). Since there existed a homology to CD4, it was proposed that LAG-3 is capable of binding to major histocompatibility complex (MHC) class II, which was confirmed by Triebel and colleagues through cell-binding assays. However, as opposed to CD4, the expression of LAG-3 takes place on more than CD4+ T cells; i.e. on myeloid cells, natural killer cells, and on activated CD8+ T cells (Lui and Davis, 2018[36]). The receptors of LAG-3 are expressed on murine plasmacytoid dendritic cells (pDC) constitutively (even though not totally confirmed) (Wu et al., 2010[70]), on a subclass of invariant NK T cells and natural killer (NK) cells (Li et al., 2017[30]; Shang et al., 2013[53]), and on activated human CD8+ (cytotoxic = CTL) and CD4+ (helper = Th) T cells, the latter of which could be detected within 24 h post *in vitro* stimulation. Moreover, the expression of LAG-3 has been detected on neurons (Wang et al., 2013[65]) and B cells (Lui and Davis, 2018[36]), though not completely validated. In addition to being expressed on membrane, LAG-3 is capable of lysosome storage, facilitating its prompt appearance on the surface of the cell following the activation of T cells (Curdy et al., 2019[7]). 

There also exists a soluble form of LAG-3 (sLAG-3), which is released through shedding at the surface of the cell, providing an extra layer of control and regulation of immunity in the TME or periphery. Presumably, sLAG-3 is capable of impairing the differentiation of monocytes in DCs or macrophages, which produces APCs that eventually suffer decreased immunostimulatory capacities (Hu et al., 2020[20]). Also, sLAG-3 has been assessed as a circulating biomarker in BC individuals who had hormone receptor (HR)-positive metastatic disease, where diagnostically detectable serum sLAG-3 had an association with a survival advantage (Wei et al., 2018[68]). Similarly, these have been found in gastric cancer recently (Duffy and Crown, 2019[12]). Together, these evidence emphasize investigating sLAG-3 as a predictive or prognostic biomarker of LAG-3-targeted therapies (Le Mercier et al., 2015[29]). The action mechanism of the lymphocyte checkpoint protein LAG-3 has always been relatively mysterious. However, it seemingly operates at least in part through the recognition and suppression of responses against MHC class II and stable complexes of peptides. Despite the fact that unknown results exist with regards to LAG-3 clinical studies, their rationale is founded on the data that suggest the co-targeting of LAG-3 as a promising strategy in order to improve the responses of immunotherapy in several human tumor types. The co-expression of LAG-3 with other molecules of immune checkpoint, such as TIM-3, PD-L1 and PD-1 is well-documented, demonstrating the promising benefits of combinatorial immunotherapies that target several TME immunosuppressive pathways could offer (Hu et al., 2020[20]). Nonetheless, it should first be demonstrated by safety data that sequentially or simultaneously combining therapies would be both tolerable and feasible. Much attention has recently been focused on LAG-3, which may belong to the second wave of immune checkpoint targets along with the receptors of TIGIT and TIM-3, as it is expressed on tumor-infiltrating lymphocytes along with the immunoregulatory receptor PD-1 and is associated with T cell exhaustion (Le Mercier et al., 2015[29]). 

### B7 family checkpoints

With respect to the perception of interplay between the immune system and cancer, recent agents have been developed in the recent decade that target B7:CD28 family checkpoints. Ever since, the capability of targeting checkpoint regulators successfully has resulted in the conductance of several clinical trials in which antibodies target the pathways attributed to the B7 family members. Members of the growing B7 family include B7-H7 (or HHLA2), B7-H6 (or NCR3LG1), B7-H5 (or PD-1H, Dies1, GI24, or VISTA), B7-H4 (or Vtcn1, B7x, or B7S1), B7-H3 (or CD276), B7-H2 (or ICOSL), B7-DC (or CD273 or PD-L2), B7-H1 (or CD274 or PD-L1), CD86 (or B7.2), and CD80 (or B7.1). It has been documented that B7 molecules are capable of providing vital positive signals for stimulating and supporting the action of T cells, as well as offering negative signals for controlling and suppressing the responses of T cells. 

Poor outcomes have been reported to be significantly associated with the expression of B7-H3 in individuals suffering from breast cancer, osteosarcoma (OS), cervical cancer, esophageal squamous cancer, gallbladder cancer, CRC, prostate cancer, lung cancer, and RCC (Ni and Dong, 2017[44]). Therefore, the expression of B7-H3 may provide an effective and feasible means for predicting the prognosis in individuals suffering from cancer. Interestingly, one of the direct targets of miR-124 in OS cells is B7-H3 (Wang et al., 2016[64]). The mRNA and protein levels of B7-H3 are decreased as a result of miR-124 overexpression, which inhibits the activity of B7-H3 3′-UTR reporter. Using miR-124 mimics to treat OS cells would enable the inhibition of the growth and invasion of cells *in vitro*, which could be abrogated through transfection with the B7-H3 expression vector. It has been suggested that miR-124 is potentially applicable as a novel onco-miRNA in OS through down-regulating B7-H3 (Wang et al., 2016[64]). 

Since the expression of B7-H7 takes place in many osteosarcoma tumors and is linked to poor survival and metastatic disease, it could be suggested that B7-H7 might enjoy a new immunosuppressive mechanism inside the tumor microenvironment of the osteosarcoma. It has been shown through the expression patterns of novel B7 family molecules that redundant mechanisms are probably used by cancers for compromising immune attack, even though unique molecules often exist, including B7-H5 and B7-H6. It could be predicted that the emphasis of immunotherapy would be laid on the effect of combined B7-H ligand. Considering the osteosarcoma tumor, the expression of B7-H3 is inversely correlated to TILs number, as well as promoting the cell invasion of osteosarcoma. Moreover, significantly shorter survival and recurrence times have been reported in patients with high expression levels of B7-H3 (Wang et al., 2013[65]). The effects of enoblituzumab are under investigation on children with B7-H3-expressing solid tumors, including desmoplastic small round cell tumors, Wilms' tumor, Ewing sarcoma, osteosarcoma, rhabdomyosarcoma, and neuroblastoma (NCT02982941). 

## Combinational Strategies for Optimizing Immune Check Point Inhibition in Osteosarcoma

Immune checkpoint blockade (ICB), which targets PD-L1, PD-1, or CTLA-4 via antibodies has resulted in unprecedented outcomes in previously incurable cancer patients (Zhao et al., 2018[72]). However, long-term benefits are observed only in some patients. For the improvement of the number of cancer types that respond and the response rate, combination therapies have emerged, which target other IRs as an instance (Curdy et al., 2019[7]). 

The members of the B7 family possess essential co-stimulatory parts in the activation of T cells. B7-1a has been reported as an alternatively spliced form of B7-1. It was demonstrated by Nagamore et al. that extrinsic (anti-CTLA-4 mAb) and intrinsic (lack of IgC-like domain in B7-1a) manipulations of the interactions of B7/CTLA-4 enhance the therapeutic efficiency of osteosarcoma vaccines that are based on B7, synergistically (Nagamori et al., 2002[42]). Both *in vivo *and* in vitro*, the efficiency of osteosarcoma-reactive CTLs has been reported to be improved significantly through blocking the interaction of PD-1/PD-L1, which results in reduced burden of the tumor as well as enhanced rate of survival in the osteosarcoma metastasis models (Lussier et al., 2015[37]). Thus, combining the blockade of the interactions of PD-1/PD-L1 and adoptive CD8+ T cell would be beneficial to pursue in the treatment of osteosarcomas. IFN-γ is capable of increasing efficacious processing of antigens for MHC-mediated antigen presentation, and thus enhancing immune responses in the tumor microenvironment (Boehm et al., 1997[4]). However, combining IFN-γ with PD-1/PD-L1 blockade is yet to be explored, as IFN-γ might be capable of simultaneously up-regulating PD-L1 expression in immune cells and peripheral tissues, and therefore suppressing the immune responses (Taube et al., 2012[59]; Wang et al., 2016[67]). As a marine-derived chemotherapeutic, Trabectedin is being used clinically for treating soft-tissue sarcomas, or combined with doxorubicin for the treatment of ovarian cancer, as a second-line single-agent treatment. However, monocytes and macrophages are also affected by the cytotoxic effects of trabectedin. Following the treatment of lung, ovarian, and fibrosarcoma tumor mouse models with trabectedin, the number of tumor site macrophages and peripheral blood monocytes were significantly reduced. It has been reported that trabectedin is capable of significantly inhibiting the growth of the primary tumor, and metastasis, of osteosarcoma through exerting effects on both tumor and immune-infiltrating cells, as well as exhibiting enhanced therapeutic efficiency in combination with PD-1-blocking antibody (Ratti et al., 2017[51]). The efficiency of anti-CTLA-4 and anti-PD-L1 antibodies was evaluated by Takahashi et al. with X-ray irradiation in both distant and local impacts on osteosarcoma. It was shown that combining anti-CTLA-4 and anti-PD-L1 antibodies (P1C4) with irradiation would enable exerting higher distant effects as well as providing greater local antitumor efficiency for osteosarcoma. Administrating P1C4 has been reported to be capable of producing a delay in the tumor growth on day 30 in 18 % of the mice. Conversely, combination therapy has been shown to have produced stronger inhibition of the tumor, both in irradiated tumor and unirradiated tumor in 67 % of the mice. Also, lung metastases were greatly reduced (by 98 %) in the combinational group, offering considerable survival benefits (Takahashi et al., 2017[58]).

## Recognizing and Managing of Immune Checkpoint Blockade Averse Events and Side Effects

Immune checkpoint blockade is responsible for a wide range of dysimmune toxicity named as immune-related adverse events (IRAEs), which include liver, gut, lung, endocrine glands, skin and other tissues. These IRAEs could possibly include the diarrhea induction or the appearance of Crohn's disease, autoimmune hepatitis, Hashimoto's thyroiditis, hypophysitis and which can potentially be life-threatening complications in case undetected or untreated (Thallinger et al., 2018[60]). It has been reported that such side effects can be recognized in 70 % of patients, though these occur in only 20-30 % after treatment with PD-L1 inhibitor (De Velasco et al., 2017[11]). It is known that rash and pruritus are the initial side effects of treatment process with anti CTLA-4 and hypophysitis, diarrhea, colitis or liver toxicity tend to occur later. After PD-L1 inhibition, gastrointestinal and cutaneous are the main IRAEs, but other phenomena such as hepatic, renal, endocrine and pulmonary are less common. Studies show that the combination of checkpoint inhibitors over the treatment procedure could potentially lead to more severe side effects, which are associated with the inhibition of CTLA-4 immune checkpoint (Thallinger et al., 2018[60]). 

It is assumed that as immunotherapy in cancer can induce the response of tumor-directed T‑cells by infiltration of T‑cells into primary tumor or related metastases, it can also lead to IRAEs in all types of tissues (Pulluri et al., 2017[50]). Treatment side effects associated with immune checkpoint blockade differ in toxicity from cytotoxic agents. In conventional cytotoxics, the toxicity time can also be delayed and not continue as a cyclical manner. The molecular mechanism of toxicity is still to be completely known, but it may be heterogeneous among individuals even with the same toxic agent (Khoja et al., 2017[25]). The hyperactivation of T lymphocytes induced by immune checkpoint inhibitors can also generate a particular response against the antigens of tumor cells, causing to the tumor suppression, though it has some side effects on health tissues known as "ontarget''. The lysis of cells by CD8^+^ T lymphocytes is responsible for releasing of different neoantigens such as tumor auto-antigens or antigens in normal tissues. The phenomenon is known as "epitope spreading'' which causes to diversification of the associated T cell repertoire and consequently suppressed immune tolerance that is worsened following of Treg inhibition. Moreover, the Th1 and Th17 lymphocytes activation induced by immune checkpoint inhibitor can increase the generation of pro-inflammatory cytokines, including IL-17 and IFN-γ. These two molecular actions are responsible for off-target toxicities (Gelao et al., 2014[16]; Yang et al., 2017[71]).

Surprisingly, it has been reported that various tumor histologies such as renal cell, melanoma or NSCLC show different irAE phenomena after treatment with PD-1 inhibitors. It was also shown that the immune cell infiltrate, tumor microenvironment, neoantigen development and the response of adaptive immune system could be impacted by histology, which is a possible explanation for different toxicities (Khoja et al., 2017[25]). 

Individuals with inflammatory disease are more sensitive to irAEs over the treatment process with the inhibitors of immune check-point. A research indicated that the pre-existing AID was considerably linked with the risk of irAEs appearance in the treated patients with anti-PD-1 antibody. Other studies also represented that the treatment of cancer with anti-PD-1 antibodies is as an effective method for AID-free patients as it is for AID patients (Danlos et al., 2018[8]). As a practical approach, anti-PD-1 and anti-CTLA-4 antibodies are entering in cancer treatment, and the number of individuals treated with such drugs will increase exponentially in the future. Therefore, it is necessary to have a close investigation on pathophysiology and molecular mechanism of autoimmune diseases. Though irAEs can be limited by using steroids, the related immunosuppression could interrupt the antitumor response (Michot et al., 2016[39]). Nivolumab and ipilimumab are two well-known drugs for melanoma treatment, which are accepted by the U.S. Food and Drug Administration, however, more than 60 % of patients indicated grade 3 or 4 related side effects. It was shown that near to 40 % of these patients are not able to continue this therapy method because of significant adverse effects (Larkin et al., 2015[28]). 

Ishihara et al. reported that the anti-cancer effect of anti-PD-L1 and anti-CTLA-4 was remarkably increased by ECM-binding addition. A further investigation showed that the PlGF-2123-144 conjugation with antibodies can increase its tissue retention. Also, a considerable side effect reduction was approved related to lower amount of antibody in plasma. The peri-tumoral injections of anti-PD-L1- PlGF-2123-144 plus anti-CTLA-4- PlGF-2123-144 can induce the tumor-infiltrating T cells, leading to the tumor suppression and increase the survival rate (Ishihara et al., 2017[21]). 

The engineering of ECM-binding antibodies is a potential approach that can be applied for effective cancer therapy by blocking immune checkpoints. Totally, those patients treated with immune checkpoint inhibitors, every possible symptom should be considered as a sign of irAE, and this should be informed to the patients. It is obvious that early detection and treatment can inhibit the appearance of 4-5 toxicities (Friedman et al., 2016[15]). 

## Predictive Biomarkers Associated with Immune Checkpoint Inhibitors

The response of immune system to immune check point inhibitors is a complicated process. Biomarkers that estimate the efficacy of immune check point inhibitors therapy and associated irAEs can be helpful in the patient selection and treatment decision-making via recognition of responders and non-responders. 

The major concern in finding predictive biomarkers is the wide range of cancer biomarker types and the genetic variation of patients (Nasiri et al., 2018[43]; Valedkarimi et al., 2017[62]). Biopsy studies of different sites of a patient indicted a considerable variation of biomarker which is associated with intratumoral heterogeneity. Advanced research will focus on development of combination biomarkers so as to estimate immune check point inhibitor therapy results and limit irAEs (Postow et al., 2018[47]). Many researches on predictive biomarkers have focused on PD-L1 overexpression, immune cell infiltration, copy number alterations, peripheral blood analyses, SNPs, neoantigen, clonality, mutational landscape, mismatch-repair deficiency and transcription factors (Darvin et al., 2018[9]).

Growing evidence show that immune inflamed tumors have high tendency to response for immunotherapy. The studies indicated that the immune inflamed tissue is more sensitive due to the activation of immune reactions and inhibition of immune evasion or suppression through ICIs immune check point inhibitors. The response of immune system to immune check point inhibitor therapy is associated with tumor-infiltrating lymphocytes (TILs) and active immune cells in the TME (Cogdill et al., 2017[6]). It was confirmed that, in the responders, tumors represent high level of neoantigen load and TILs, particularly effector cells, low levels of MDSC, high ratio of Teff to Treg, and raised secretion level of IFN-γ or other cytokines. However, in the non-responders, the TME includes high rate of immunosuppressive cells, like, MDSCs and Tregs, and very low rate of activated lymphocytes and NK cells (Darvin et al., 2018[9]; De Angulo et al., 2008[10]; Simeone et al., 2014[57]). 

Furthermore, since PD-L1 has an important role in suppression of immunogenicity and is known as a potential target for PD-L/PD-L1 antibodies, it was considered as a predictive biomarker for such treatments. The individuals with PD-L1-positive tumors can achieve more advantage from PD-l/PD-L1 antibodies compared with those patients with PD-L1-negative tumors (Duffy and Crown, 2019[12]; Wei et al., 2018[68]). A meta-analysis study which included 8 prospective randomized clinical trials (involved 4174 patients) with different form of cancer assessed the value of PD-L1 evaluation for predicting benefit from PD-1/PD-L1 (Shen and Zhao, 2018[56]). This study showed that the use of PD-1/PD-L1 inhibitors can remarkably increase the survival rate in both PD-L1 negative (HR, 0.80; 95 % CI, 0.71- 0.90) and PD-L1 positive [hazard ratio (HR), 0.66; 95 % CI, 0.59 - 0.74] compared to conventional chemotherapy. However, the PD-1/PD-L1 antibodies were more efficient for the patients with positive PD-L1 rather than negative PD-L1 (Duffy and Crown, 2019[12]; Shen and Zhao, 2018[56]).

In a group of patients with advanced metastatic sarcoma, a study reported various partial responses in osteosarcoma, dedifferentiated chondrosarcoma and epithelioid sarcoma after treatment process with nivolumab (Paoluzzi et al., 2016[45]). Another study, however, revealed that the treatment of synovial sarcoma with anti-CTLA-4 antibody could not induce the immunological antitumor responses in the studied patients (Maki et al., 2013[38]). Therefore, identification of biomarkers is a main step to improve the selection process of sarcomas, which can respond to the treatment based on immune checkpoint (Veenstra et al., 2018[63]). 

Recently, the hyperprogression phenomena was explained in a group of patients treated with anti-CTLA-4 antibodies and anti-PD-1/PD-L1. In some cases, the growth of tumor was increased after treatment of these inhibitors. This phenomena is mainly associated with the mouse double minute (MDM) 2 and MDM4 gene amplification and possible EGFR mutations (Champiat et al., 2017[5]; Kato et al., 2017[24]). The MDM family members have been documented as key regulators of cancer protective responses. The appropriate function of the MDM2 and MDM4 family is critical for normal development of breast tissue, but also for stabilizing genomic fidelity. As it is shown increased levels of the MDM2 and MDM4 genes are associated with breast cancer (Haupt et al., 2017[18]). In a comprehensive statistical study, it was shown that MDM2 amplification can be detected in most of sarcoma cases (Momand et al., 1998[41]). The amplification of 12 chromosomes (12q13-15 region) which includes MDM2 gene is a potential hallmark of parosteal osteosarcoma, and well-differentiated and dedifferentiated liposarcoma. Also, some other sarcomas indicate MDM2 amplification at a very low rate in cancer cells, including malignant peripheral nerve sheath tumors and conventional osteosarcomas. These researches propose that the treatment of sarcoma subtypes with the inhibitors of immune checkpoints must be carefully evaluated (Aleixo et al., 2009[3]; Veenstra et al., 2018[63]). 

## Conclusion

Cancer immunotherapy has now turned into an efficacious therapeutic approach as a result of the elucidation of the role of immune checkpoint inhibitors in the activation of host immune responses. Selective anti-PD-L1, anti-PD-1, and/or anti-CTLA-4 mAbs have brought about a revolution in treatment of many types of cancer, among which lung cancer and melanoma have seen the greatest clinical efficacy. However, not in all patients is immunotherapy fully effective, and thus selecting biomarkers is required to optimize the treatment. With regards to OS, immunotherapy has been less effective. So far, despite the promising results from preclinical studies, solid evidence supporting the efficacy of immunotherapy in these patients has not been provided. Better understanding the molecular mechanisms defining the OS immune competence is required for developing predictive biomarkers as well as effective combination approaches, including chemotherapies or co-stimulatory and targeted agents. One of the main challenges in this regard would be the identification of patients with particular tumor and tumor infiltrating stroma functional and molecular features, among the heterogeneous OS spectrum, which could be treated by immunotherapy effectively.

## Notes

Vafa Meftahpour and Ali Aghebati-Maleki contributed equally as first author.

Elham Safarzadeh and Leili Aghebati-Maleki (Immunology Research Center, Tabriz University of Medical Sciences, Tabriz, Iran; Tel: (+98) 413 3364665, Fax: (+98) 41 3364665, E-mail: leili_aghebati_maleki@yahoo.com) contributed equally as corresponding author.

## Declaration

### Authors' contributions

Vafa Meftahpour and Ali Aghebati-Maleki conceived the idea and provided the draft of the manuscript. Ali Fotouhi participated in literature survey. Elham Safarzadeh and Leili Aghebati-Maleki provided inputs for the design and the final edition of the article. Leili Aghebati-Maleki critically revised the manuscript. All authors read and approved the final manuscript.

### Funding

This study was supported by Research Vice-Chancellor at Tabriz University of Medical Sciences, Iran [Grant No. 68036].

### Declaration of interest

The authors declare that they have no conflict of interest.

## Figures and Tables

**Table 1:**
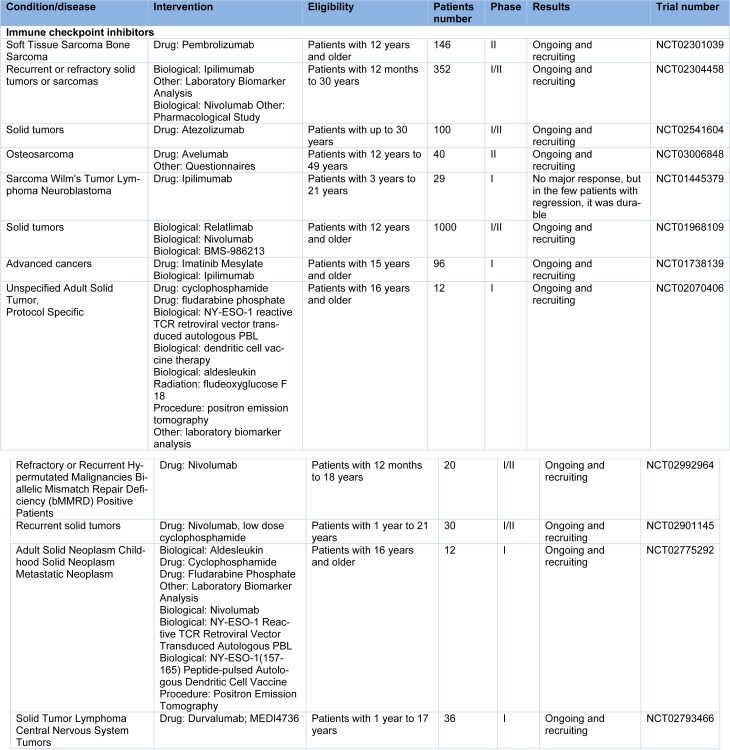
Clinical trials for osteosarcoma by checkpoint inhibitor
